# The existential dimension of the experience of seclusion: a qualitative study among former psychiatric inpatients

**DOI:** 10.1186/s12888-023-05208-7

**Published:** 2023-10-03

**Authors:** Eva S. Trapman, Arjan W. Braam

**Affiliations:** 1 Spiritual Care, Custodial Institutions Agency, Ministry of Justice and Security, The Hague, The Netherlands; 2 Department of Humanist Chaplaincy Studies for a Plural Society, University of Humanistic Studies, Utrecht, The Netherlands; 3 Department of Emergency Psychiatry, Department of Residency Training, Altrecht Mental Health Care, Utrecht, The Netherlands

**Keywords:** Seclusion, Existential meaning, Ultimate concerns, Boundary situations, Spiritual experiences

## Abstract

**Background:**

Seclusion is a coercive measure - temporary confinement in an almost empty, non-stimulating room in a closed psychiatric admission ward to prevent (further) urgent danger due to a mental disorder. Although there is observational research about patients’ behaviors during separation (e.g. hitting walls or doors, sleeping, or praying), research into the subjective and existential dimension of the experience of seclusion in psychiatry is rare.

**Aim:**

Aim of the current study is to describe and analyze - using the theoretical lenses of Yalom (1980) and Jaspers (1919) - how clients experience their involuntary stay in a seclusion room in a closed psychiatric clinic in existential terms.

**Methods:**

A qualitative study was carried out among former clients (N = 10) who were asked, in retrospect, about their existential concerns in the seclusion room. In the thematic analysis, the main, deductive codes were theory based (Yalom, Jaspers), composed of subcodes that were inductively derived from the interviews.

**Results:**

The respondents affirmed the ultimate existential concerns about death (e.g. sensing to be dead already), lack of freedom (e.g. loss of agency), isolation (e.g. interpersonal, not able to speak, feeling an object) and meaninglessness. With respect to the latter, the respondents reported a rich variety of spiritual experiences (both negative, such as knowing to be in hell, as positive, hearing/imagining a comforting voice or noticing/imagining a scenery of nature in the room).

**Discussion:**

Although some experiences and behaviors may conflate with symptoms of psychosis, the participants generally expressed a relief about the ability to talk about their experiences. Sharing and discussing the existential experiences fits into the paradigm of psychiatric recovery and personalized care. Their intensity was obvious and might have warranted additional support by a chaplain or spiritual counselor in mental health care settings.

**Supplementary Information:**

The online version contains supplementary material available at 10.1186/s12888-023-05208-7.

## Introduction

Separation is a coercive measure - temporary confinement in an almost empty, non-stimulating room in a closed psychiatric admission ward. The following criteria apply: there must be danger, for the client himself or for others, as a result of a mental disorder, that cannot be averted in any other way [3]. The seclusion measure today has a necessary, but also problematic character in contemporary psychiatry.

Seclusion can offer safety and the opportunity to regain control, but it also has its downsides: often inducing fear and the experience of personal disqualification. Research has now shown that separation can be harmful to clients both physically and emotionally [14]; traumatic [9, 19], and can also damage the treatment relationship [21, 27]. In addition, separation is related to stigmatization and is a severe expression of power inequality.

People can go through major life crises during psychiatry admissions, with separation being a possible tragic nadir. Although there is not yet one specific typology available in psychiatry for these crises of existence, existential questions do arise at those moments [4, 5].

Within the existing research on separation the perspective of the clients remains virtually unnamed: little information is available about the existential side of clients’ experiences before, during and after seclusion [11, 27, 20]. Ethical challenges seem to be at the core of the seclusion practice [10]. Van der Laan, Rietveld and De Boer [16] describe how a group of adolescents experienced their seclusions. The research focuses on the observation with regard to the stay in the seclusion room, but this is done *from* a treatment perspective^1^. Namely, the study evaluates the legal requirements that must be met in the case of involuntary seclusions: efficiency, proportionality and subsidiarity. The criterion of subsidiarity is subjective, because what is least intrusive for one client can have a major impact on another. The researchers conclude that the client experience is the only measure of subsidiarity.

Van der Merwe and colleagues [21] provide an overview of mental health nurses’ reports on behavior in the seclusion room. However, observing and reporting on the behavior of secluded patients does not provide any information about the *​meaning​* of this behavior. Seven American papers report on behavior and six of them describe behaviors such as sleeping, laying down and praying [7, 15, 28]. Here, the descriptions are confined to observations of visible behavior, but the literature does not mention what the behaviors implied. Three papers describe destructive behavior and the hitting of walls, windows and doors [7, 28]. Other behavior in the seclusion room as described in these papers includes walking (naked) up and down, crying, urinating, name-calling, doing sports or exercises and self-harm. Hence, these are again descriptions from outside: the patients are observed but do not speak themselves. Apart from reports of narratives of personal recovery, systematical research into the subjective, existential dimension of seclusion experience does not exist or is in any case scarce.

Van der Merwe and colleagues [21] show that the people who end up in a seclusion room may be experiencing severe psychological disturbances and are at the end of their ‘common’ coping capabilities. In the seclusion room most material sources of meaning, support or comfort/consolation are absent. Possibly immaterial, to the extent of intangible sources such as spirituality (for a definition, see [22]) may therefore come to the fore. It is not possible to isolate someone from his or her spirituality. One’s spirituality may therefore be that which ‘remains’ in the separation.

In the present study, the term ‘existential’ contains three components: the appearance of existential themes/ultimate concerns [31], to what extent the experience can be described as a ‘boundary situation’ [13] and what role spirituality played within these existential and terminological frameworks.

Aim of the current study is to describe and analyze - using the theoretical lenses of Yalom [31] and Jaspers [13] - how clients experience their involuntary stay in a seclusion room in a closed psychiatric clinic in existential terms. The current study addresses the following research questions:

1. In what way are existential concerns experienced during an involuntary stay in a seclusion room?
2. To what extent were the seclusion admissions experienced as existential boundary situations?

### Existential themes: ultimate concerns

Yalom [31] distinguishes four *ultimate concerns* that every human being inevitably has to deal with in his or her life: death, lack of freedom, isolation and meaninglessness. Yalom’s conceptual approach with four main types of existential concerns was guided by a rich knowledge of literature on existential issues from several scientific traditions, such as existential psychology and philosophy. Furthermore, he applied his clinical experience with psychotherapy groups of clients diagnosed with cancer. Within clinical psychiatry, the four prototypical categories of existential concerns have not been regularly used. The themes may have a different emphasis in clinical psychiatry. With respect to death and dying, questions may relate to the prospect of recurrent episodes, further suffering, loss of health, loss of mental health or even suicide. Furthermore, in mental illness episodes, one may have less control over one’s own decisions and even be subjected to the unavoidable decisions of others, e.g. imposing involuntary admission or treatment or involving guilt, shame, and/or victimhood clearly mirroring a lack of freedom. This may also give rise to questions about a lack of personal competence and identity. Mental illness episodes can also lead to a profound sense of isolation caused by symptoms of the disorder, e.g. alienation from oneself, others, society as a whole, misunderstandings and stigmatization. Finally, facing a mental illness may arouse a need to make sense of the suffering with the core questions about meaning and meaninglessness: Why? Why me?

### Boundary situations

Less well-known but equally relevant in exploring existential experiences is Karl Jaspers’ [13] theoretical work on boundary experiences. This has served as one of the sources of inspiration for Yalom’s existential model. In Table 1: *Yalom and Jaspers: A theoretical comparison between ultimate concerns and boundary situations*, we give a concise theoretical comparison between the four ultimate concerns and the four boundary situations. Jaspers described a boundary situation as a situation in which a confrontation takes place with one’s own total vulnerability in the face of suffering and death, struggle, guilt and chance.

**Table 1:** Yalom and Jaspers: A theoretical comparison between ultimate concerns and boundary situations

Ultimate concerns (Yalom, 1980)	The character of an ultimate concern	Boundary situations (Jaspers, 1919)	The character of a boundary situation
	It is possible to reflect on it, an attitude can be developed to deal with the *ultimate concerns.*		It is acute and overwhelming, it can be called an *Erschütterung der Seele.*
Yalom: Death	Death in all forms; death as (abstract) given.	Jaspers: Death	Death as an overwhelming, inescapable threat or a deep realization of its existence.
Yalom: Freedom	Humans have the freedom, and with it the responsibility, to shape their own life.	Jaspers: Guilt	The inevitable guilt that humans incur as a result of their (non-)action and life.
Yalom: Isolation	A person is ultimately alone in the world.	Jaspers: Struggle	The struggle for the right to exist and the space to exist.
Yalom: Meaninglessness	Humans have to create their own meaning. In boundary situations, on the other hand, there are no images and narratives to give meaning.	Jaspers: Chance	The precariousness of fate. The opposite of necessity, causation, purpose or meaning.

**Death** as a boundary situation can be understood as the realization of the temporariness of all that lives (general aspect) and the unimaginable and radical aspect of one’s own end of life (individual aspect) [5]. The boundary situation of **guilt** means the unavoidable guilt that humans incur as an irreversible result of our action or non-action and life. The third boundary situation is the **struggle** for the right to exist and the space to exist. In forming one’s own identity, in love and in the relationship with others [5]. Finally, **chance** as a boundary situation refers to the vicissitudes of fate - the opposite of causation, purpose or coherence. We find chance in (apparently) unjust and unnecessary events. Events that can raise the urgent question: ‘why is this happening to me?’ A boundary situation entails a fundamental low in one’s life. A situation in which it is strongly experienced that there is a life before and a life after this moment. There is something crushing about boundary situations: “(…) those who experience it cannot help but feel that a roof of heaven is tearing open here, under which we usually live our lives” [12]. However, experiencing a boundary situation can lead to new attitudes towards life [13].

## Method

### Procedure and sample

The study population consists of former clients who once had undergone involuntary placement in a seclusion room during psychiatric admission. The respondents were selected according to two criteria: (1) the respondent had spent one or more times in a seclusion room in a closed psychiatric admission ward in the Netherlands and (2) this stay (or stays) was/were involuntary. No selection was made on the duration of separation, which ranges from two days to five weeks. Also, no selection was made on the various grounds on which it was decided to separate them, which range from suicidality to aggression to psychosis (see Table 2: *Overview respondents*). Furthermore, no selection was made with respect to how long ago the separation had occurred. This varies from 1992 to 2020 (see Table 2). The first three respondents were recruited through public sites for Mental Health Recovery projects and the next seven were found through snowball sampling. All respondents signed a document of informed consent prior to their participation. The study was approved according to the methodological and ethical standards and regulations of the University of Humanistic Studies.

**Table 2:** Overview respondents

	Sex	Age	Number of stays in seclusion room	Duration of longest stay	Time past since last stay	Religious (R) or spiritual (S) background	Stated alleged background/reason for seclusion
1	F	35–40	5	3–5 days	1–2 years	R - S -	danger to self (suicidality), not psychotic
2	M	55–60	3	1–2 weeks	15–20 years	R + (Catholic) S +	confused or psychotic
3	F	40–45	1	3–5 days	2–5 years	R - S -	confused or psychotic and therefore danger to self
4	F	45–50	1	3–5 days	15–20 years	R - S +	confused or psychotic and therefore a danger to the environment/others
5	M	45–50	3	10–15 days	5–10 years	R - S +	confused or psychotic and therefore a danger to the environment/others
6	M	40–45	1	3–5 days	5–10 years	R - S -	confused or psychotic and therefore a danger to the environment/others
7	F	35–40	1	3–5 days	5–10 years	R - S -	confused or psychotic
8	M	35–40	8	1–2 days	1–2 years	unknown	confused or psychotic and therefore a danger to self and/or the environment/others
9	F	45–50	2	3–5 weeks	15–20 years	R - S +	danger to environment/others
10	F	20–25	1	5–10 days	1–2 months	unknown	confused, danger to self (suicidality), not psychotic

### Data collection

Data was collected by conducting ten semi-structured in-depth interviews. The topic list contained questions about general characteristics of the seclusion(s) (how often, when, for how long), followed by questions about the experience. General questions about the experience were raised first, then questions about coping - what did you do to get through difficult moments? Subsequently, it was probed to which extent the participant had a religious or spiritual outlook regarding the stay in the seclusion room, to facilitate further discussion of meaningful experiences later in the interview. Then came questions about boundary situations and the four ultimate concerns. Follow up questions were asked about what was brought to the table with every topic. Probing questions were based on: what did that experience mean to you?

The interviews were conducted in Dutch. A member check was offered to all respondents. Two respondents made use of this and verified the transcripts.

### Data analysis

The transcripts of the interviews were coded using the Direct Content Analysis method [30], a data analysis approach that starts with a deductive approach, then combines it with an inductive strategy.

First, the transcripts were deductively coded using a code list consisting of pre-determined codes, including the four existential themes of Yalom [31] and the four boundary situations of Jaspers [13].​ As a result, the theoretical framework is closely adhered to throughout the analysis. This first round was done by the first author. In the second round of the analysis inductive coding was used, which allowed the creation of new codes. This way, relevant data that fall outside the predefined frameworks, could appear. This is done in close cooperation with the second author, who verified the formulation of subcodes and thought along with ambiguous codes. In this second round, the existing codes could also be further specified into different subcodes (see: Table 3. *Data analysis*). For example, the pre-determined code ‘Guilt’ includes the subcodes: ‘failure’, ‘punishment’ and ‘responsibility’. The codes are presented in code trees (see *Appendix: Code tree*).

**Table 3:** Data analysis

Step 1: Deductive coding	Step 2: Inductive coding (open and axial)
Pre-determined codes:	
Yalom (1980): • Death • Lack of freedom • Isolation • Meaninglessness Jaspers (1919): • Death • Struggle • Change • Guilt The theoretical framework is closely adhered to throughout this step of the analysis. *This step was done by the first author.*	A second round of inductive coding allowed new codes to appear. This is done through a first phase of open coding, followed by a second phase of axial coding. Also, existing codes could be further specified into different sub codes. *This step is done in close cooperation with the second author, who thought along with ambiguous codes and the formation of sub codes.*

Reference: Staa, A. van & de Vries, K. (2014). Directed content analysis: een meer deductieve dan inductieve aanpak bij kwalitatieve analyse. KWALON 2014 (19) 3

Boundary situations have a shattering, absolute character. At the same time, speaking about boundary situations has a very high abstract and subjective character and it is therefore a complicated matter to interpret concrete experiences as such. First of all, we looked at whether the respondents speak of an ultimate low or turning point. We also looked at whether respondents felt that a break in time had occurred: a life ‘until’ the seclusion, and a life ‘after’ the seclusion.

Before addressing existential themes and boundary situations, some main patterns of the reported behaviors during seclusion are described. Because existing research only describes behavior, a brief overview is given of not only the behavior but also some insights into the meanings of their activities, as well as an impression of their helpful or non-helping function.

## Results

### Behavior in seclusion and its meaning

The behaviors and actions of the respondents during their seclusions can be divided into four categories. The first category concerns actions aimed at making contact, the second concerns creativity, and the third consists of (self)destructive activities. Finally, there is a fourth category of other notable actions.

Actions in the first category include: having contact with family, other people or nature. The second category, creativity, include drawing (6 respondents), writing (5), singing (4), dancing or rocking (3), crafts (1), reading (2) and making music (2). The meanings that respondents give to these are: finding something to hold on to, it gave encouragement, reminded of positivity, it expressed things that cannot be put into words, it kept the brain occupied, brought structure, relaxed the body, discharged emotions and helped to get through time, it made them tired ‘until nothing else remains’ and a few hoped to communicate something with it to the care providers. The activities of both these categories were usually helpful for the respondents.

The third category, the (self)destructive activities, includes: various forms of self-injury (in 7 respondents), hunger strike (1) and breaking furniture (2). Underlying meanings behind inflicting the self-injury were: expressing general protest, dealing with unrest or despair, seeking a high, moving a feeling to something physical, attempting to communicate with care providers, striving for privacy, do something very concrete to stay in reality and the search for stimuli because of boredom. Respondents indicated that these activities were sometimes helpful and calmed them, but most of the time they were not.

The latter category includes: praying or confessing (5 respondents), actively expressing anger (3) and trying to escape from the separation (5). Finally: one respondent tells how she smeared her own menstrual blood on her lips. This was not well noticed and because it involved blood, this act was immediately seen as the result of self-injury. However, she indicates that she did this (albeit in a very confused state) because she was thirsty and had nothing to drink and therefore moistened her lips.

### Existential themes: Ultimate concerns

#### Death

Death is a present Ultimate Concern (UC) in the experiences of seclusion (39 times). The UC of death appears in three ways. The first is ‘the fear of dying’, three respondents make this more specific: e.g. the fear of being killed by the nursing staff. The second subcode is ‘the fear of already being dead’, four respondents experienced this.

And I was really convinced that I had died, in my delusion. And [name nurse] woke me up. And he said: ‘[respondent’s name], no, you’re not dead - I am real, and we’re going to have breakfast, it’s not true, you’re alive’. But I had really been so far away, I really thought I was dead. And who says it isn’t. That I’ve been somewhere where people come who have died (4:40).

Finally, three respondents talk about suicidal thoughts or actions during the separation. Two respondents express a contrasting feeling. For the first respondent the seclusion brought some calm. She struggles with suicidality in her daily life. The second respondent calls the seclusion life-saving. ‘I think if they hadn’t put me in the seclusion room that last time, it might have gone wrong for me. Then we might not have been able to have this conversation now’ (9:38).

#### Lack of freedom

Lack of freedom is an important theme in the seclusion experiences (81 times). The sense of freedom is seriously compromised in most stories. The feeling of being unable to do anything, of not being seen and heard and of not having a say in the matter predominates.

Two opposite subcodes have emerged: ‘powerlessness’ and ‘empowerment’ (see *Appendix: Ultimate Concerns Code tree*). Powerlessness appears as physical constraint and loss of control and agency.

Some respondents managed to focus on what *was* still possible within this setting. Referring to small but empowering things within the subcode ‘empowerment’, such as consistently ringing the bell in the hope of making contact, or asking for a drink when thirsty. Other empowering aspects came up in the powerful wish to break free: ‘I have to get out of this, and I’m going to do everything I can to do that (4:5)’. The feeling of being able to shape one’s own life is clearly sharpened in the seclusion experiences. For example by creating a different, imaginary, friendlier world. One respondent tells how a black square was painted on the wall of the seclusion room. This black square, she imagined, was a kind of portal that led to a nicer place: ‘a world in which I understood the things’:

An idealistic world, in which everything just - I wanted to get out of there so badly - that it was really there, that it could be real, that I wanted to believe that, that I could escape for a while. (…) it was like I was lying in the grass, and I um, it was all right, and my horse was there, and my cat was there, and my parents were there, and there was a sun… so it was just really, a world that didn’t exist, but that I wanted, needed at the time. (10:7)

#### Isolation

Isolation is the most assigned code (157 times) and is also referred to as the most essential theme. The most obvious finding to emerge from this code is the profound experience of loneliness. Within this experience, we distinguish three subcodes representing different kinds of loneliness: stressful loneliness, existential loneliness and loneliness by mental dysfunctioning. Stressful loneliness is the most frequent subcode. Loneliness by mental dysfunctioning (inability due to mental dysfunction such as sedative medication or psychosis) the least frequent one. The physical environment of the seclusion room is mentioned as an intensifier of the experiences of stressful loneliness as well as loneliness by mental dysfunctioning. The environment was barren, strange and often incomprehensible to the respondents, partly because of the condition they found themselves in. For example, because there were no ‘normal’ stimuli. No normal behavior could be triggered, one respondent explains. For this respondent, if there had been a normal toilet instead of the ‘cardboard hat’ that she didn’t recognize as a toilet, she would have been less confused about what was expected of her. She was driven further and further from reality by the alienating environment.

A contrasting experience was also reported: one respondent said that he felt locked up so often that the seclusion did not limit him that much, on the contrary in fact. ‘(…) I feel trapped so many times already. Locked up in my own body, so in the end the separation is, yes… that can be quite liberating, so to speak’ (07:26).

An experience of existential loneliness is the following: ‘The isolation cell is that corner in the kitchen that you cannot reach with the mop and where the dirt remains. That’s how I felt. I’m just the filth of society, in some very remote place’ (4:26).

Several respondents indicated that they were not or hardly able to communicate with the nurses. Some of them were physically unable to speak or walk, which led to the feeling of loss of control and agency. One respondent tells how she was no longer able to talk, but knew that she was able to write. She kept signaling to the camera and to the nurses with hand signals: ‘talk - no, write - yes’.

In addition to the poignant descriptions of disconnection, *positive* examples of connectedness that did exist have also been mentioned. One respondent tells how a nurse started dancing with her. This gave the respondent a basic, physical recognition, she had a dance lesson history and can clearly remember that she thought: ‘hey, I know this’. She calls it a little ‘recovery moment’. Other examples of alternative communication mentioned are: lovingly washing hair; the silent presence of a nurse who came to read a newspaper in the seclusion room; exchanging glances through a window with a cleaning lady; knocking on the wall with the neighbor in another room; finishing each other’s songs with the same neighbor.

The fourth subcode of ‘Isolation’ is ‘object/dignity’. This includes experiences of feeling humiliated and not feeling seen or heard. ‘You just feel like your human dignity is being taken away. As if you are an object. You are being tucked away’ (1:12).

#### Meaninglessness

There are examples of lack of meaning and there are examples of prominence of meaning. *Lack of meaning* manifested itself mainly in questions about the meaning of the seclusions. Most respondents did not understand where they were and why they were there. Why am I here? What kind of crazy place is this? What is expected of me? And when they did know where they were, the question arose regularly: what’s the use of this? For some, this question was bigger: does my life still have meaning? One respondent said that prior to the separation she had little hope for the rest of her life. ‘Actually, I already realized that my life was never going to be anything, I guess’ (1:20). The seclusion was a confirmation of this idea. ‘Just lock me up, that’s what I’m worth’ (1:23).

*Prominence of meaning* is reflected in what the respondents call religious or spiritual experiences. For example, unity experiences occur, but also the experience of having contact with God or an angel. Variations of the spiritual behaviors of prayer and confession were found in the stories of five respondents. Eight out of ten respondents describe different spiritual experiences and behaviors (see Table 4: *Spiritual experiences and behaviors*). One respondent tells about the experience of having died and being taken back to life by a nurse. Reflecting on this he tells:

**Table 4:** Spiritual experiences and behaviors

1	Praying
2	Visit of an (later turned out to be) imaginary pastor / the strong feeling of being helped
3	Guardian angel visit
4	An experience of primal power
5	An appointment with God
6	A conversation with God
7	Feeling the presence of God / a divine spark
8	Picking up a rose at the bottom of the well
9	An experience of unity / essence consciousness / deep acceptance
10	A unity experience
11	The experience of dying and being recalled
12	Wanting to reach heavenly light in the ceiling
13	Confessing for several hours ‘about everything I had done wrong in my life’
14	Being in hell
15	The feeling of being helped (by deceased people, birds, butterflies)
16	Meaningful connection to a big tree

I was very emotional. Yes. (…) I can still feel it, you know. I’m almost going to cry because it feels so strong, that I now also realize that he just really put a kind of ground under me with warmth and love. That’s why I didn’t fall through. He caught me. (…) I will be eternally grateful to him for that. (…) And deeper than this I could not fall. It’s best if you can die and then you live. Then you are in your extra time. Yes. (4:42)

Four different respondents talk about meaningful visits, one from a pastor (while afterwards no one had been with her), one from a guardian angel and two respondents experienced contact with God. ‘And then I came up with those life questions such as: will this ever be okay again? And then a voice just popped into my head: yes, now go to sleep, it’s okay. And that to me was the voice of God’ (9:24). Another example is the ‘day-long conversation with God’ about which a respondent tells. He was convinced he was dead and never felt closer to God than he did then, he says. The main meaning of these experiences is to generate consolation or reassurance. Another story contrasts with this: a respondent tells he has had the experience of being in hell.

Yeah, then I really had an experience like I was in hell, and then I heard the most horrible things, and voices that said the most horrible things, and… Yeah, I heard some kind of creatures walking around, some kind of wild boar, a kind of sound a wild boar makes. I heard that walking around, they let them in other people’s cells, I heard them and they then ate them (5:10).

Regularly, shyness or shame occurred while the respondents talked about spirituality. This was noticeable by statements such as: ‘This may sound very psychotic, but…’. One respondent explicitly says that he finds it more difficult to talk about prayer than about the seclusion: ‘because I actually think I am crazier because of praying than that I was in the seclusion’ (7:28).

Some experiences were frightening, but in general it can be concluded that the respondents cherish their spiritual experiences. The most common function of the spiritual experiences and behaviors is to seek comfort or reassurance. Connectivity with nature was also a source of comfort for two respondents. Praying usually gave a feeling of reassurance. The sense of being connected to something greater - nature, the unity of all living things or the divine - softened the isolation somewhat.

### Boundary situations

#### Death

Death as a boundary situation means: experiences in which death was or felt very close to the respondent. This code was given 39 times. Death as a boundary situation therefore has three subcodes, which connect to the three themes of death as ultimate concern: ‘Thinking to be dead already’, ‘Death near / fear of being murdered’ and ‘Not knowing how to live on’.

An example of a boundary situation around death that comes close to a possible spiritual experience is the already mentioned ‘day-long conversation with God’ about which a respondent tells. He was convinced he was dead and never felt closer to God than he did then, he said.

Finally, several respondents were afraid of being murdered in the seclusion room. Death felt concrete and close.

At one point I heard they were setting fire to the cells around me. Then I heard people screaming and then I thought yes, I’ll be one of the next where they do that. (5:10)

#### Guilt

One respondent thought he was in hell, and that the seclusion was his punishment for all the mistakes he ever made in life. This is the only experience around guilt, that we consider a boundary situation. Three other respondents share feelings of guilt, connected to three subthemes: ‘failure’, ‘punishment’ and ‘responsibility’. To them, these feelings were an additional source of stress, but this did not have the crushing character of a boundary situation.

#### Struggle

The seclusion experiences are characterized by struggle. Struggle is the most striking and present theme encountered in the experiences of separation. The code was assigned the most of the four boundary situations. Subcodes are: ‘the right to exist’, ‘endurance’ and ‘acceptance of fate’.

‘Struggle’ often overlaps with the code ‘death’. In some interviews the battle for the right to exist is the central topic. One respondent’s story is particularly marked by physical coercion. She describes the moments when she was sedated by five staff members and her pants and underpants were pulled down. She felt thrown back to her core and in that moment had a sense of everything she had experienced in her life. An example of ‘acceptance of fate’ in response to the struggle for existence:

I don’t fight, and you can destroy me completely. And what is, that is. And even if I’m dead, I’m still somewhere - then there’s peace, acceptance… or wherever I am, I’m not part of it. That compulsion is not mine, it is not mine, it is yours. You bear that responsibility to rule over me, as a different human being. And I’m not going to resist anymore. (2:52)

#### Chance

Chance is rarely mentioned by the respondents. They reflected on the question how it could have come to this, along with questions such as: how is it possible that I am here? Should this have happened or not? Apart from these reflections, which thematically do have to do with chance, chance *as a boundary situation* cannot really be identified in the stories.

### The boundary situation as a turning point in the life course

To interpret these above experiences in terms of ‘positive’ or ‘negative’ would do them a disservice. Nevertheless, among three respondents a positive upward movement can be detected in their lives after the seclusion admissions. One of them aptly describes this as picking up a rose from the bottom of a dark well. Another changed course after the seclusion admission and started living a much healthier life. The third speaks of an admission where he experienced empowerment in addition to a lot of suffering. In the seclusion he came to realize that he had to take his place in the world again. We can conclude that three out of ten respondents consider the seclusion as a turning point or wake up call that has turned their lives for the better. However, for three others who also saw it as a turning point, it took on an opposite meaning: the seclusion was seen as confirmation of one’s supposed worthlessness, or the trust in finding help and a sense of hope had been definitively less than before the seclusion.

## Conclusion

Aim of this study is to describe and analyze how clients experience their involuntary stay in a seclusion room in a closed psychiatric clinic in existential terms, by asking two research questions.

Research question 1. In what way are existential concerns experienced during an involuntary stay in a seclusion room?

The respondents affirmed the four ultimate existential concerns: death, lack of freedom, isolation and meaninglessness. With respect to the latter, the respondents report a rich variety of spiritual experiences, either supportive and helping or associated with psychopathological symptoms. Or both, which is in line with the ‘both/and’ perspective of Ouwehand et al. [25], who describes religious experiences among patients, of whom 42% see these experiences as ‘both spiritual and pathological’.

The respondents were not selected on experience with spirituality in the seclusion room. That is, there was nothing about spirituality in the call for recruitment. However: eight respondents talked about spiritual experiences. Looking at these findings, it is important to recognize that attention for meaningful experiences can be helpful to the respondents. To some, the experience has changed their view of the world.

Research question 2. To what extent were the seclusion admissions experienced as existential boundary situations? Some seclusions have been experienced as boundary situations, interpreted as both extremes on the spectrum from ‘lifesaving’ to ‘traumatic’.

## Discussion

### Interpretation of the results

#### Ultimate concerns

In the present study, respondents reflected about their subjective and existential dimension of their psychiatric seclusion experience in the past. They clearly affirm that existential concerns - death, lack of freedom, isolation and meaninglessness [31] - arise during their seclusions. Death is an evident topic in the seclusion experiences. The conflict that Yalom describes between the fear of dying and the will to live, is visible. Isolation is the most commonly assigned code and also the most essential, describing the total loss of control and agency and different types of loneliness. Yalom distinguishes three types of isolation: intrapersonal -, interpersonal - and existential isolation. Our subcodes stressful loneliness and existential loneliness confirm his descriptions, whereas our subcode ‘loneliness by mental dysfunctioning’ describes a new type that falls outside Yalom’s theory. It may be the case that some of our respondents were more highly sedated or psychotic or anxious during the seclusion, and the impact this had on their experience of seclusion is profound. These quotes, as well as the quotes that we coded as ‘object/dignity’, seem related to the idea of objectification as explored by Jean Paul Sartre in 1943. According to Sartre, without personal interaction, and therefore without realizing an interpersonal bodily self, reflection on one’s own self is no longer constructed [8]. The reflective self falls away, leaving only a certain physical self. The remaining object has thus become a disconnected object, sometimes even an abject object, in which integral reflection is less feasible, especially due to the concurrent mental dysfunction.

In addition to the poignant descriptions of disconnection, positive examples have also been mentioned of connectedness that did exist, for example in the form of alternative communication. Meaninglessness is reflected, albeit somewhat less strongly than the other three themes. Moreover, there are examples of prominence of meaning, where spiritual experiences are to be found.

#### Boundary situations

Some seclusions have been experienced as boundary situations, especially pertaining to ‘death’ and ‘struggle’. The seclusion experiences have been interpreted as both extremes on the spectrum from ‘lifesaving’ to ‘traumatic’. Six respondents indicated that the seclusion was a turning point, but in the form of a low that has done more harm than good. The interviews with at least three respondents show that the seclusion was experienced as a kind of ultimate turning point, whereby the seclusion is referred to as a ‘wake up call’. Experiencing a boundary situation can lead to the development of new attitudes towards life [13]^2^. This is indicated by the stories of these three respondents.

### Spirituality

#### Discussing spirituality in treatment contact: a recommendation

The respondents in the sample expressed a certain relief about their sharing of existential concerns and even spiritual experiences during the interviews. The intensity of the experiences was obvious and might have warranted additional support by a chaplain or spiritual counselor in mental health care settings. Braam and Verhagen [6] have the impression that patients rarely bring up their experiences with regard to spirituality in the treatment contact. Ouwehand’s [25] research into spiritual experiences in people with bipolar disorder also shows that patients prefer not to discuss these with their psychiatrist. Leget [18] points out that it is generally not easy to talk about spiritual experiences. Indeed, almost all respondents mentioned discomfort or embarrassment about spirituality in connection with treatment contact. At the same time, most indicated that they had a need for this. This is in line with the aforementioned work by Saad and Madeiros [29].

A recommendation for nurses, clinicians and therapists would therefore be to invite patients to talk about these concerns and experiences. In a recent study among mental health care patients, patients had the preference that care professionals would introduce subjects like religiousness, spirituality or matters of meaning in life [23]. Also Ouwehand and colleagues [24] described care preferences of patients with bipolar disorder regarding religious and spiritual experiences. This pertained for example to non-judgemental listening, but in some cases also to finding a critical sounding board. Nevertheless, the expected or actual lack of interest of clinicians had made several participants reluctant to discuss their experiences in treatment settings [24]. From an ethical point of view, one may however question whether treatment staff on closed psychiatric wards is in the right position to start these conversations. Questions with respect to existential issues would not benefit from any impression of being forced. Therefore, although the careneed to share about existential issues is clear, the timing when to introduce the subject is uncertain. This question about timing and role of treatment staff there requires further inquiry.

#### Religious and spiritual coping

When someone experiences certain events as stressful, an appeal is made to the resources he has to deal with them. Pargament [26] has added a third category to Lazarus and Folkman [17] distinction of two categories of coping: meaning-focused coping, which also includes religious and spiritual coping. The function of spiritual coping is reflected in some of the experiences. Coping behaviors carry an active component. Some experiences - such as praying, confessing, making an appointment with God - show this active character. Other spiritual experiences seem to be received, they have come to them (‘receptive coping’, [1].

#### The ambiguous distinction between spiritual experiences and psychopathology

The distinction between authentic spiritual experiences and psychopathology is by no means unambiguous [6]. ‘How can one distinguish between strange experiences (anomalous experiences) and actual psychotic experiences?’ ( [6], p. 19). From a medical perspective spiritual experiences can in some cases be seen as pathological [25]. In the present study, some of the experiences can be recognised as symptoms of psychosis. For example, the Cotard delusion turns up: a conviction that one or more somatic organs or even the entire body already died. Furthermore, the inability to speak for one other respondent was probably the symptom of mutism, as can occur during catatonic states. In addition, the feeling of being locked up in one’s own body can also result from antipsychotic medication, especially when the dosage is high and the patient has no experience with accommodating to its effect.

Following on from Ouwehand [25], this study recognizes that such experiences may both be pathological and meaningful, rather than one or the other. A both/and approach: the religious experience can be understood from the perspective of psychopathology, but also from the context of meaning-making. Religious/spiritual experiences and psychiatric symptoms can therefore co-exist [29].

### Limitations

One limitation of the present study relates to the small scale of the sample and thus of the data set. Interviewing ten respondents provides a limited picture for describing the existential dimension of seclusion experiences. An attempt was made to speak to a diversity of respondents, in age, background, from different parts of the country and in terms of diagnosis. That was partly successful. The diversity is quite large in some areas, but there is a bias with regard to diagnosis. Among the respondents, the diagnosis of bipolar disorder and, to a lesser extent, personality problems were common. This means that there is an underrepresentation of, for example, schizophrenia. People with for example highly paranoid experiences may be less able to tell and are less likely to participate in a study like this one. Replication or the study is needed, in a sample of patients who are diagnosed with schizophrenia. Another limitation of the study is that only clients who were secluded in a closed psychiatric admission ward in the Netherlands were approached. No other sectors of care, such as forensic settings, or countries were included.

Concerning the method of this study, the data are based on what former inpatients remember from their time in the seclusion room, and the fact that for some it was a long time ago (See Table: *Overview respondents)* may have colored the memory and this affects the reliability of the experiences told. Secondly, the data are based on what they decided to tell us when they answered open questions about their experience of the seclusion. What happened on the spot is unknown. The present study describes experiences, only in retrospect. However, since this study is descriptive, interpretive and has an exploratory character, it is assumed that people give meaning to phenomena and that by exchanging them one arrives at a jointly constructed reality [4]. In this form of research, we wanted to know how the people we study interpret their situation, ‘attempting to make sense of, or interpret, phenomena in terms of the meanings people bring to them’ ( [4], p. 26).

The present study explores and describes existential and spiritual experiences during the stay in the seclusion room. We have cautiously stated that some of the experiences are being interpreted as boundary situations. An option for further research might be to look how the existential experiences of seclusions are integrated in the life course. In that way it will be more clear whether the experiences indeed represented boundary situations, because it can then be placed in the life course and it is not viewed as an isolated event.

### Electronic supplementary material

Below is the link to the electronic supplementary material.

Supplementary Material 1: Appendix A: Ultimate Concerns Code tree; Appendix B: Boundary Situations Code tree

## Data Availability

Data available on well-motivated request due to restrictions, e.g., privacy or ethical. The data are not publicly available due to the concern of keeping respondents’ anonymity.

## Footnotes

1 The ‘treatment perspective’ encompasses the reasons for seclusion, from the perspective of care providers and practitioners (Liégouis, 1996). The ‘experience perspective’ includes the client’s experience (Liégouis, 1996).

2 “Es bleibt, wenn einmal die Grenzsituation erreicht ist, nur die Reaktion in der Entwicklung neuer Kräfte, die ihren Ausdruck wohl in Formeln finden, aber eigentlich Lebenseinstellungen, Lebensgesinnungen sind” (Jaspers, 1919, p. 232).
