# Supplementary material for: The existential dimension of the experience of seclusion: a qualitative study among former psychiatric inpatients
Source: BMC Psychiatry. 2023 Oct 3;23:715. doi: 10.1186/s12888-023-05208-7 (PMC10548759; doi:10.1186/s12888-023-05208-7)
Supplement: Supplementary file 1 — Supplementary Material 1: Appendix A: Ultimate Concerns Code tree; Appendix B: Boundary Situations Code tree [file 12888_2023_5208_MOESM1_ESM.docx]

**Appendices***Appendix A: Ultimate Concerns Code tree*

**Death** [ultimate concern]
 The fear of dying
 The fear of being dead
 Suicide

**Lack of freedom** [ultimate concern]
 Powerlessness
 Empowerment

**Isolation** [ultimate concern]
 Existential loneliness
 Stressful loneliness
 Loneliness (by mental disfunctioning)
 Object/dignity

**Meaninglessness** [ultimate concern]
 Lack of meaning
 Prominence of meaning
 Spiritual experiences
 Spiritual behaviors

*Appendix B: Boundary Situations Code tree*

**Death** [Boundary situation]
 Thinking to be dead already
 Death near / fear of being murdered
 Not knowing how to live on

**Guilt** [Boundary situation]
 Failure
 Punishment
 Responsibility

**Struggle** [Boundary situation]
 The right to exist
 Endurance
 Acceptance of fate

**Chance** [Boundary situation]
 Randomness of fate
 Necessity

**Turning point**
 New attitudes towards life
